# Supplementary material for: Prevalence of burnout syndrome in Brazilian anesthesiologists during the COVID-19 pandemic: A cross-sectional survey
Source: PLoS One. 2025 Feb 18;20(2):e0313538. doi: 10.1371/journal.pone.0313538 (PMC11835280; doi:10.1371/journal.pone.0313538)
Supplement: S1 File — (PDF) [file pone.0313538.s001.pdf]

APPENDIX 1

SOCIO-DEMOGRAPHIC AND PSYCHOSOCIAL QUESTIONNAIRE

This questionnaire is part of the research intitled “Prevalence of Burnout Syndrome in Brazilian Anesthesiologists during the COVID-19 Pandemic: a Cross-Sectional Survey” and aims to obtain information about your activity and daily life as an Anesthesiologist.

Guidelines:

a) Please answer all items without exception;

b) Your answers should reflect the truth;

c) Remember: your privacy will be preserved, according to the Informed Consent previous signed;

d) This information is for research purposes only.

1- AGE: ________; DATE OF BIRTH: /. /. ;

2- SEX: MALE( ) FEMALE( );

3- PLACE OF ORIGIN: ___________________________;

4- MARITAL STATUS:

SINGLE( )

MARRIED( )

DIVORCED( )

WIDOW( )

DOMESTIC PARTNERSHIP( )

5- DO YOU HAVE CHILDREN:

YES( ); IF YES, HOW MANY? ________

NO( )

6- YEARS OF ANESTHESIA PRACTICE, INCLUDING RESIDENCY: _______;

7- NUMBER OF HOSPITALS/LOCATIONS WHERE YOU WORK: _________;

8- HOW MANY HOURS DO YOU WORK PER WEEK? _____________;

9- DO YOU WORK AT NIGHT?

YES( )

NO( )

10- DO YOU WORK ON WEEKENDS?

YES( )

NO( )

11- DO YOU LIVE WITH:

FAMILY( )

FRIEND/COLLEAGUE( )

ALONE( )

OTHER( )

12- HAVE YOU EVER CONSIDERED GIVING UP YOUR SPECIALTY?

YES( )

NO( )

13- HOW MANY HOURS PER WEEK DO YOU DEVOTE TO LEISURE ACTIVITIES? ________;

14- DO YOU BELIEVE THAT RESPONDING TO THE MASLACH BURNOUT INVENTORY DURING A COVID-19 PANDEMIC MAY HAVE INFLUENCED YOUR QUESTIONNAIRE RESPONSES? PLEASE INDICATE THE OPTION THAT MOST CLOSELY REFLECTS HOW YOU FEEL.

a) Yes, and I believe I am or may be experiencing burnout syndrome due to the pandemic.

b) Yes, and I probably rated my responses more pessimistically because I answered the questionnaire during the pandemic.

c) Yes, but I don't believe the pandemic is affecting me so negatively.

d) No, answering the questionnaire during the pandemic did not affect my responses.

e) No, and I even believe I rated my responses more optimistically because I answered the questionnaire during the pandemic.
