# Supplementary material for: Prevalence of burnout syndrome in Brazilian anesthesiologists during the COVID-19 pandemic: A cross-sectional survey
Source: PLoS One. 2025 Feb 18;20(2):e0313538. doi: 10.1371/journal.pone.0313538 (PMC11835280; doi:10.1371/journal.pone.0313538)
Supplement: S2 File — (PDF) [file pone.0313538.s002.pdf]

PLOS ONE

# PREVALENCE OF BURNOUT SYNDROME IN BRAZILIAN ANESTHESIOLOGISTS DURING THE COVID-19 PANDEMIC

*Liana M T A Azi, Thaiane S Ferreira, Thiago Cerqueira-Silva, Luis A S Diego, Marcos A C Albuquerque, Matheus L Azi*

## CROSS SECTIONAL SURVEY

### 19,6% OF BURNOUT SYNDROME

### 56,5%% OF HIGH RISK FOR BURNOUT

## STATISTICS

**48,2%**
≥ 27 in Emotional Exhaustion

**36,4%**
≥ 10 in Depersonalization

**36,9%**
≤ 33 in Reduced Personal Accomplishment

**Having considered giving up the specialty was the most strongly related factor**

**OR 4.72**
(95% CI 3.30-6.83)

**To adress the high prevalence it is recommended to**

- Enhancing Work Environment
- Providing Continuous Psychological Support
- Promoting Mindfulness and Stress Management Techniques
- Implementing Comprehensive Mental Health Programs
- Conducting Longitudinal Studies

## PROTECTION FACTORS

**Leisure time of more than 21 hours per week**
74% less
(OR: 0.26 (95% CI 0.12-0.52))

**Anesthetic practice between 16 and 25 ys**
60% less
(OR: 0.40 (95% CI 0.16-0.99))

**Having a child**
41% less
(OR: 0.59 (95% CI 0.36-0.96))

**Working in the Northeast region of Brazil**
39% less
(OR: 0.61 (95% CI 0.37-0.99))

**When participants were asked whether the pandemic had influenced their responses,**

**59%** reported no effect
