# Supplementary material for: Prevalence of burnout syndrome in Brazilian anesthesiologists during the COVID-19 pandemic: A cross-sectional survey
Source: PLoS One. 2025 Feb 18;20(2):e0313538. doi: 10.1371/journal.pone.0313538 (PMC11835280; doi:10.1371/journal.pone.0313538)
Supplement: S3 File — (PDF) [file pone.0313538.s003.pdf]

Table: Multivariable Analysis of Factors Associated with Burnout Syndrome and High-Risk Development.

		Burnout		High risk	
Variables		OR (multivariable)	p-value	OR (multivariable)	p-value
Age	< 30 yr	-		-	
	30 - 39 yr	1,21 [0,68;2,18]	0,517	1,56 [0,85;2,89]	0,152
	40 - 49 yr	2,08 [0,85;5,08]	0,107	1,70 [0,73;4,02]	0,221
	> 50 yr	2,48 [0,70;8,69]	0,157	2,28 [0,76;6,95]	0,145
Sex	Male	-			
	Female	0,93 [0,65;1,34]	0,712	1,74 [1,28;2,36]	<0,001
Marital status	Married	-		-	
	Single	1,17 [0,68;2,02]	0,571	1,10 [0,65;1,87]	0,718
	Other	0,93 [0,57;1,51]	0,78	0,83 [0,55;1,25]	0,369
Children	No	-		-	
	Yes	0,59 [0,36;0,96]	0,035	0,75 [0,49;1,13]	0,173
Live with	Friends/ Family	-		-	
	Alone	0,65 [0,38;1,09]	0,107	1,35 [0,81;2,24]	0,249

Region	Southeast	-		-	
	Northeast	0,61 [0,37;0,99]	0,049	0,49 [0,33;0,71]	<0,001
	North	0,94 [0,39;2,10]	0,878	1,21 [0,55;2,76]	0,634
	Federal District	0,78 [0,28;1,92]	0,613	0,48 [0,22;1,03]	0,061
	Center-west	1,62 [0,87;2,96]	0,120	1,06 [0,57;2,00]	0,859
	South	0,78 [0,49;1,23]	0,292	0,61 [0,41;0,91]	0,014
Hours of leisure per week	≤ 5	-		-	
	6 to 10	0,64 [0,44;0,94]	0,021	0,69 [0,47;0,99]	0,044
	11 to 20	0,36 [0,20;0,62]	<0,001	0,37 [0,24;0,58]	<0,001
	> 21	0,26 [0,12;0,52]	<0,001	0,31 [0,19;0,50]	<0,001
Years of anesthetic practice	< 5	-		-	
	5 -15	0,62 [0,36;1,06]	0,080	0,54 [0,31;0,91]	0,023
	16 - 25	0,40 [0,16;0,99]	0,045	0,38 [0,17;0,85]	0,020
	> 25	0,29 [0,08;1,04]	0,054	0,30 [0,10;0,85]	0,024
	<45	-		-	

**Hours of work per week**	46 to 60	1,13 [0,65;2,00]	0,664	1,32 [0,87;2,01]	0,189
	> 60	1,47 [0,84;2,61]	0,183	2,35 [1,52;3,65]	<0,001
**Night time shifts**	No	-		-	
	Yes	1,17 [0,70;1,98]	0,566	1,19 [0,79;1,79]	0,414
**Work at weekends**	No	-		-	
	Yes	0,83 [0,44;1,62]	0,58	1,08 [0,67;1,75]	0,742
**Thought about quit the specialty**	No	-		-	
	Yes	4,72 [3,30;6,83]	<0,001	4,99 [3,70;6,77]	<0,001
