# Supplementary material for: Prevalence of burnout syndrome in Brazilian anesthesiologists during the COVID-19 pandemic: A cross-sectional survey
Source: PLoS One. 2025 Feb 18;20(2):e0313538. doi: 10.1371/journal.pone.0313538 (PMC11835280; doi:10.1371/journal.pone.0313538)
Supplement: Attachment — Submitted filename: Response to Reviewers.pdf [file pone.0313538.s005.pdf]

Prevalence of Burnout Syndrome in Brazilian Anesthesiologists during the COVID-19 Pandemic: A Cross-Sectional Survey

Response to Reviewers

Dear Editor and Reviewers,

We would like to extend our gratitude for the thorough and insightful feedback provided on our manuscript. Your detailed comments and suggestions have been invaluable in guiding our revisions, and we believe that our work has been significantly improved as a result.

We have carefully considered each point raised and have made the necessary adjustments to enhance the clarity, robustness, and overall quality of our study. To facilitate your review, the modifications and adjustments suggested by Reviewer 1 are highlighted in yellow, and those suggested by Reviewer 2 are marked in green within the text.

In addition, to enhance the dissemination of our findings, we have created an infographic that visually summarizes the key details of our study. This infographic is intended for use on social media platforms such as Instagram and Twitter, making the results more accessible to a broader audience.

We hope that the revised manuscript now meets the high standards of PLOS ONE and addresses all the concerns raised. Thank you for your constructive input and for the opportunity to refine our work.

The authors

Reviewer #1 (Highlighted in Yellow)

Comment 1: The manuscript does not add new information to the existing body of knowledge on burnout syndrome in anesthesiologists. The findings closely mirror those of previous studies, including a recent study published in the Brazilian Journal of Anesthesiology, which contradicts the authors' claim on page 16 that “This was the first study to assess this important and current topic of burnout among all Brazilian anesthesiologists and anesthesiology residents during the COVID19 pandemic”. Please see this article: [https://www.ncbi.nlm.nih.gov/pmc/articles/PMC9371977/]. Authors

should emphasize unique aspects of the study, or consider a more novel angle or detailed subgroup analysis to enhance the manuscript's contribution to the literature.

Response: We appreciate the reviewer bringing this important point to our attention. We would like to clarify that our study differs significantly from the referenced study in several key aspects:

1. **Questionnaire Used:** Our study utilized the Maslach Burnout Inventory (MBI), which is widely recognized as the gold standard for assessing burnout. In contrast, the referenced study employed the Oldenburg Burnout Inventory (OLBI). The MBI is specifically designed to measure three dimensions of burnout: emotional exhaustion, depersonalization, and personal accomplishment. Its comprehensive approach is highly regarded in the literature for its reliability and validity in measuring burnout, particularly in healthcare professionals (Maslach et al., 1996).
2. **Population Studied:** The referenced study focused solely on anesthesiology residents and gathered 205 responses. Our study, however, encompassed the entire population of Brazilian anesthesiologists, including both residents and practicing anesthesiologists, resulting in over 1000 responses. This broader scope allows for a more comprehensive and representative analysis of burnout prevalence across different stages of professional development and work settings.
3. **Scope and Scale:** By including a larger and more diverse sample, our study provides a more detailed and capilarized panorama of burnout prevalence among Brazilian anesthesiologists during the COVID-19 pandemic. This extensive dataset enables a nuanced understanding of burnout across various subgroups, which was not possible in the referenced study.

To address the reviewer's suggestion, we have emphasized these unique aspects in the revised manuscript and conducted additional subgroup analyses to further highlight the distinctive contributions of our study.

Comment 2: Data Collection Dates: The manuscript does not provide exact dates of data collection, which is crucial for contextualizing the findings, especially considering the rapidly evolving situation of the COVID-19 pandemic.

Response: Thank you for your valuable feedback. We have clarified the data collection process in the Methods section to indicate that the questionnaires were sent on three specific dates according to the availability of the mailing list from the Brazilian Society of Anesthesiology. The exact dates of data collection, which were already included in

the Results section, are: 21 June 2021, 17 January 2022, and 16 September 2022. This addition should provide a clearer context for our findings and their relevance to the evolving situation of the COVID-19 pandemic.

Comment 3: 3. STROBE Guidelines: The STROBE guidelines were not fully reported in the manuscript. Specific elements are inadequately covered.

Response: Thank you for your observation. We have carefully reviewed the manuscript to ensure full adherence to the STROBE guidelines. Specific elements that were inadequately covered have now been addressed and incorporated. Additionally, we have attached the completed STROBE checklist as a supplementary file to the revised manuscript. We believe these updates will enhance the transparency and rigor of our study.

Comment 4: • Methodology: The methodology section lacks clarity regarding the sampling method and participant recruitment. Clarify whether the sampling size calculations followed the standard procedure.

Response: Thank you for your feedback. We have revised the methodology section to provide more clarity regarding the sampling method and participant recruitment. Specifically, we have elaborated on how the sample size was determined and the recruitment process.

Comment 5: Statistical Analysis: The manuscript mentions the use of logistic regression but does not provide sufficient detail on the model selection process and the handling of potential confounders. Consider adding a more thorough explanation of the statistical methods used.

Response: Thank you for your constructive feedback. We have expanded the Statistical Analysis section to provide more detail on the model selection process and the handling of potential confounders.

> “For the outcomes of burnout and high risk for burnout, we performed univariable analyses using the $\chi 2$ test. Variables with a possible association to either outcome ($p < 0.1$ in univariable analyses) were included in separate multivariable models for burnout and high risk for burnout. In addition to the variables with $p<0.1$, we also included age and sex in all models, as these variables are well-established confounders in burnout research.

Binary logistic regression was used to calculate the odds ratios (OR) and 95% confidence intervals (CI) for each outcome. The confidence intervals were utilized to interpret the strength of the associations, providing a measure of precision and reliability. For all statistical analyses, a two-tailed P-value < 0.05 was deemed statistically significant."

Comment 6: Ethical Considerations: While ethical approval is mentioned, the manuscript should detail the ethical considerations, such as informed consent and data confidentiality, more explicitly.

Response: Thank you for your feedback. We have added more explicit details regarding the ethical considerations in the manuscript. Specifically, we have clarified the process of obtaining informed consent and the measures taken to ensure data confidentiality. Additionally, the informed consent form has been included as Supplementary Material 3, and we have added a section titled "Ethical Considerations" in the Methods.

Comment 7: Discussion and Conclusion: The discussion section should better integrate the findings with existing research. Highlight any inconsistencies or confirmatory findings with previous studies and discuss potential reasons for these. • The conclusion should offer more specific recommendations for practice and future research.

Response: Thank you for your insightful feedback. We have revised the Discussion and Conclusion sections to better integrate our findings with existing research, highlight any inconsistencies or confirmatory findings, and offer more specific recommendations for practice and future research. The modifications are clearly highlighted in yellow in the revised manuscript. Additionally, we have added a new section on recommendations based on the current literature to enhance the practical implications of our study.

Reviewer 2 (Highlighted in Green)

General Feedback: The authors should be congratulated in continuing the work already carried out in this important area of collaborative research ie burnout syndrome in

anesthesiologists. The aim of this study (according to the abstract) is: to assess the prevalence of burnout syndrome in Brazilian anesthesiologists during the COVID-pandemic. The method used was an online survey conducted during the years of the pandemic obtaining a convenience sample of respondents. Psychometric instruments and statistical methods used seem appropriate. The authors report but also conclude that burnout is highly prevalent in Brazil. General feedback: Overall, the paper is well written and contributes to a large body of (anesthesia) literature where prevalence of burnout is assessed. The connection with COVID is interesting for the anesthesia community. However, a few issues should be addressed. It feels that the connection between the research question (burnout during the Covid pandemic) vs the conclusion drawn (the prevalence found) is missing.

Response:

Dear Reviewer,

Thank you for your encouraging feedback and for acknowledging the importance of our research on burnout syndrome among anesthesiologists during the COVID-19 pandemic. We appreciate your recognition of the methods used and the relevance of our findings. We understand your concern regarding the connection between the research question and the conclusion.

We have revised the manuscript to strengthen the linkage between the impact of the COVID-19 pandemic and the observed prevalence of burnout. This includes a more detailed discussion on how the pandemic has influenced the working conditions and stress levels of anesthesiologists, contributing to the high prevalence of burnout. These revisions aim to provide a clearer narrative connecting the unique challenges posed by the pandemic to the mental health outcomes observed in our study.

Comment 1: In the introduction the authors hypothesize that there might be an increase in burnout due to increased work stress. This is reasonable and interesting. Data was indeed collected during the pandemic. The conclusion drawn (abstract and discussion) that burnout is prevalent is self-evident; it is basically a re-statement of your results. Therefore: where is the connection to the research question?

Response: Thank you for your insightful feedback. We understand the importance of clearly connecting our research question regarding the impact of the COVID-19 pandemic on burnout among anesthesiologists with our conclusion. We have revised the manuscript to better articulate this connection. Specifically, we have expanded the discussion section to include a detailed analysis of how the unique stressors and working

conditions during the pandemic have contributed to the high prevalence of burnout observed in our study. This revision aims to provide a more explicit linkage between the pandemic-related factors and the mental health outcomes reported.

Comment 2: For example, how does this found prevalence relate to the hypothesis/idea of increase in burnout because of the pandemic? Is it higher/lower than expected? Why?

Response: We appreciate the reviewer's insightful question regarding the relationship between the found prevalence of burnout and the hypothesis of an increase due to the COVID-19 pandemic. As discussed in our manuscript, the impact of COVID-19 on the work environment and burnout among anesthesiologists has been a critical area of concern. The prevalence of burnout found in our study did not show a significant increase, which was somewhat unexpected given the high stress and increased workload associated with the pandemic.

Several factors may contribute to this finding. First, anesthesiologists possess a diverse skill set that allows them to adapt to various healthcare settings beyond the operating room, such as the ICU and emergency departments. This versatility may have provided a buffer against the increased stress, preventing a sharp rise in burnout rates. Second, our study's timing might be a crucial factor. Participants reported their experiences relatively early in the pandemic, and the full impact on their mental health and professional self-realization might not have manifested completely at the time of data collection. Third, institutional support and resilience-building initiatives might have played a role in mitigating the expected increase in burnout.

It is important to acknowledge that while our findings did not reveal a significant increase in burnout, this does not diminish the potential long-term effects of the pandemic on anesthesiologists' mental health. Continuous monitoring and further longitudinal studies will be essential to comprehensively understand the pandemic's impact on burnout over time.

We have highlighted these points in the revised discussion section (marked in green) to provide a clearer connection between our findings and the hypothesis regarding the pandemic's effect on burnout.

Comment 3: Three invitations to respond have been sent according to the results section. (by the way, this should be in the methods section.) Have the authors considered comparing burnout rate between those three groups?

Response: Thank you for your observation. We have moved the information about the three invitations to respond to the methods section, creating a new subsection titled "Participant Recruitment" to ensure clarity and proper organization of our manuscript.

Regarding the comparison of burnout rates between the three groups, we acknowledge that this specific analysis was not conducted in our study. The primary focus of our research was to assess the overall prevalence of burnout among anesthesiologists during the COVID-19 pandemic. Comparing burnout rates between the groups based on the timing of their responses could provide additional insights into temporal variations in burnout levels. However, our study design and sample size were not optimized for this specific type of subgroup analysis.

We agree that comparing these groups could be valuable and recommend it as a potential area for future research. Conducting such analyses in future studies could help to identify any temporal trends in burnout rates and provide a deeper understanding of how different phases of the pandemic may have influenced the mental health of healthcare professionals.

We have included a note in the discussion section (highlighted in green) to acknowledge this point and suggest it as a direction for future research.

Comment 4: Have the authors compared their findings to figures from before the pandemic? A quick literature search shows several papers reporting burnout levels in Brazilian anesthesiologists (eg Sousa, Ana Rafaela Campos, and Joana Irene de Barros Mourão. "Burnout em anestesiologia." Revista Brasileira de Anestesiologia 68 (2018): 507-517.)

Response: Thank you for your pertinent question. In our literature review, we identified a systematic review conducted by Sousa et al. (2018), which included 45 articles on burnout in anesthesiology. However, none of these studies specifically examined Brazilian anesthesiologists. Notably, two of the 45 articles were authored by a Brazilian, but these studies were conducted within the North American population, not within Brazil. To the best of our knowledge, the only study that evaluated burnout among Brazilian anesthesiologists during the COVID-19 pandemic focused on anesthesiology residents rather than anesthesiologists of all ages (dos Santos, et al. 2023). This study highlighted the burnout risk among anesthesiology residents during the second wave of COVID-19, providing valuable insights but within a different demographic.

Therefore, a direct comparison with figures from before the pandemic for Brazilian anesthesiologists is not feasible due to the lack of specific pre-pandemic studies within

this population. Our study aims to fill this gap by providing current data on burnout among Brazilian anesthesiologists, which can serve as a reference for future research and comparisons.

We have incorporated this explanation into the revised discussion section (highlighted in green) to clarify the context of our findings and the limitations regarding comparisons with pre-pandemic data.

Comment 5: Related to the former: Why do the authors compare their figures with those found in Zambia? As the authors state, the working conditions are very different compared to those in Brazil.

Response: Thank you for your thoughtful question. The comparison of our figures with those found in Zambia was intended to provide a broader context for understanding the variability of burnout levels in different global settings. We acknowledge that the working conditions in Zambia are indeed very different from those in Brazil. However, including such comparisons can offer valuable insights into the diverse factors that contribute to burnout across different healthcare environments.

Burnout is a multifaceted issue influenced by various factors, including workload, resources, healthcare infrastructure, and cultural attitudes towards work and stress. By comparing our findings with those from other countries, we aim to highlight the global nature of the burnout problem and emphasize that it is not confined to a single region or set of circumstances. This approach helps to underscore the need for tailored interventions that consider the unique challenges faced by healthcare professionals in different settings.

We have clarified this rationale in the revised discussion section (highlighted in green) to ensure that the purpose of these comparisons is well understood and to emphasize the importance of considering a wide range of contexts when addressing burnout in healthcare.

Comment 6: The background section in the abstract is okay. However, could the authors reflect on the statement that "Among medical specialties, anesthesiology is considered one of the most stressful"? This is indeed repeated in anesthesia literature, but is it supported by evidence? Are there studies where several medical specialties and their stress are compared?

Response: Thank you for your insightful comment. We acknowledge that the statement regarding anesthesiology being one of the most stressful medical specialties is commonly found in the literature. While there are several studies that highlight the high levels of stress and burnout among anesthesiologists, we recognize the importance of supporting such claims with comparative evidence across different medical specialties.

In response to this comment, we have revised the abstract to reflect a more evidence-based perspective. Additionally, we have reviewed the literature for studies comparing stress levels among various medical specialties. For instance, a study by Shanafelt et al. (2012) compared burnout and satisfaction with work-life balance among physicians in various specialties, revealing that some specialties, including emergency medicine and general surgery, also report high levels of burnout and stress. However, anesthesiology consistently ranks among the top specialties in terms of burnout prevalence.

Based on this, we have revised the abstract (Highlighted in green)

Comment 7: Most of the introduction is taken up by explanation of burnout syndrome, only at the end a line of thought becomes apparent. Perhaps the statement of the "problem" should be first and would the paper gain by a reworking. (the background section in the abstract works better in that respect in my opinion)

Response: Thank you for your valuable suggestion. We agree that presenting the problem statement earlier in the introduction would improve the clarity and focus of our manuscript. We have restructured the introduction to emphasize the main problem from the beginning and to streamline the flow of information. The revised introduction now clearly states the issue of burnout among anesthesiologists during the COVID-19 pandemic and outlines the objective of our study earlier. The detailed explanation of burnout syndrome has been condensed and integrated into the broader context of the study.

Comment 8: Anesthesiology, in particular, stands out for its ability to promote high stress levels. This is true, of course, however and related to question 8: is anesthesia special in that respect? And why?

Response: Thank you for your insightful question. Anesthesiology is indeed considered a particularly stressful medical specialty, and there are several reasons for this distinction. Anesthesiologists are responsible for the perioperative care of patients, including the management of vital functions during surgery, which requires constant

vigilance and rapid decision-making. The high-stakes nature of their work, where errors can have immediate and severe consequences, adds to the stress levels.

Several studies have highlighted these unique stressors. For example, anesthesiologists often face long hours, irregular schedules, and the necessity to respond to emergencies, which can contribute to physical and mental exhaustion. Additionally, the need to manage complex patient cases and the pressure to maintain patient safety under challenging conditions further exacerbates stress. The combination of these factors creates a high-stress environment that is somewhat unique to anesthesiology compared to other medical specialties.

We have included a brief explanation in the revised introduction to address this point and provide context for why anesthesiology is particularly prone to high stress levels.

Comment 9: In the method section some elements seem to be missing. For example, what are the psychometric properties of the instrument used? Which were the procedures used to select the variables to be included in the adjusted model?

Response: Thank you for pointing out these important aspects. We appreciate the opportunity to clarify and expand upon the methods used in our study. We have updated the methods section to include details on the psychometric properties of the Maslach Burnout Inventory (MBI) and the procedures used for variable selection in the adjusted models.

Psychometric Properties of the Maslach Burnout Inventory (MBI): The MBI is a widely used instrument for assessing burnout, with extensive validation across various populations, including healthcare professionals. The MBI-Human Services Survey (MBI-HSS) used in this study consists of 22 items measuring three dimensions of burnout: emotional exhaustion (EE), depersonalization (DE), and personal accomplishment (PA). The reliability of the MBI has been demonstrated in numerous studies, with Cronbach's alpha coefficients typically ranging from 0.70 to 0.90 for the subscales. The validity of the MBI is supported by its strong correlations with related constructs such as job satisfaction, organizational commitment, and mental health outcomes.

Variable Selection for Adjusted Models: Variables were initially selected for inclusion in the multivariable models based on the results of univariable analyses, with a p-value threshold of <0.1. This approach allowed us to identify potential predictors and confounders. Age and sex were included a priori in the final models due to their well-established roles as confounders in burnout research. The final models were developed using stepwise regression, balancing model fit and complexity. This method iteratively

adds and removes variables to identify the most parsimonious model, ensuring that only variables that significantly contribute to the model are retained.

We have incorporated these details into the methods section, highlighted in green.

Comment 10: Perhaps not all non-Brazilian anesthesiologists understand the significance of working in the Northeast region of Brazil. Perhaps some clarification?

Response: Thank you for highlighting this important point. We apologize for not providing sufficient context about the significance of working in the Northeast region of Brazil in our initial manuscript. The Northeast region has distinct geographical and socio-economic characteristics that can impact the work environment and quality of life for anesthesiologists. We have now included additional context in the manuscript discussion to clarify these aspects.

Comment 11: Table 1: perhaps an explanation in the legend of the table would clarify which comparisons are made and how they reached significance.

Response: Thank you for your valuable suggestion. We have updated the legend of Table 1 to include an explanation of the comparisons made and how significance was determined (highlighted in green).

Comment 12: Figure 3: concerning the multivariate logistic regression, in the adjusted model. What are the characteristics of this model? How much variance in burnout is explained by these factors? What does this mean?

Response: Thank you for your insightful questions. We appreciate the opportunity to provide additional details regarding the multivariable logistic regression model presented in Figure 3.

Characteristics of the Model: The variables included in the multivariate logistic regression model were selected based on the univariable analyses between each variable and the outcome (burnout / high risk), using the a p-value threshold of <0.1, in addition to age and sex that were included regardless of p-value in the univariable analysis, due to well established role as confounders in the burnout research Of all variables collected in the survey, only two variables weren't included in the final model

for Burnout (Number of Hospital affiliations and live with family/friends or alone) We have rewritten the paragraph in the results section to make it clearer:

> "In the logistic regression model for assessing the association with burnout, the following variables were included age, sex, have children, years of anesthetic practice, hours of work per week, night time shifts, work at weekends, hours of leisure per week, thoughts about quitting the specialty, geographic region, marital status All variables demonstrated a statistically significant association with burnout in the univariable analysis, except for sex (p-value = 0.25)."

Variance Explained by the Model: The amount of variance in burnout explained by the factors included in the model was assessed using Nagelkerke's R^2, which is a pseudo R^2 measure appropriate for logistic regression models. In our model, Nagelkerke's R^2 was 0.24, indicating that approximately 24% of the variance in burnout can be explained by the factors included in our model. Regarding high-risk for burnout, the Nagelkerke's R^2 was 0.35.

Meaning of the Results: The results of the multivariate logistic regression indicate that several factors significantly contribute to the likelihood of experiencing burnout among anesthesiologists. Key findings include:

- Anesthesiologists with children had a lower likelihood of experiencing burnout (OR: 0.59, 95% CI: 0.36-0.96).
- Longer leisure time was associated with a reduced likelihood of burnout, with those reporting more than 21 hours of leisure time per week showing a 74% lower chance of experiencing burnout (OR: 0.26, 95% CI: 0.12-0.52).
- Anesthesiologists practicing for 16-25 years had a 60% lower chance of experiencing burnout (OR: 0.40, 95% CI: 0.16-0.99).
- Working in the Northeast region of Brazil was associated with a 39% lower chance of burnout (OR: 0.61, 95% CI: 0.37-0.99).
- Having considered quitting the specialty was the strongest predictor of burnout, with an OR of 4.72 (95% CI: 3.30-6.83).

The model highlights the complexity of burnout and underscores the importance of addressing multiple dimensions to mitigate this issue effectively. We have updated the manuscript to include these details and improve the clarity and comprehensibility of our findings.

Comment 13: I feel the discussion could be improved by a reworking. Perhaps the format of starting with the main findings, then discussing those from the perspective of

the body of literature, then discussing the meaning/implications of the findings. Then of course discussing the study limitations, and an explanation why in your paper, this does not influence the conclusion. And then an overarching conclusion: I miss that here.

Response: Thank you for your suggestion. We have restructured the discussion to follow the format you recommended. The revised discussion now begins with the main findings, discusses these findings in the context of existing literature, explores their implications, and addresses study limitations. We have also added a conclusion to summarize the key points and implications of our study.

Comment 14: For the reader, to see the same elements returning in both introduction, methods, results, and discussion provides a good experience. Several important variables associated with burnout are discussed, however without a real introduction elsewhere. Why were they important to examine in the first place? How do they connect to the research question? Eg. This was the first study to assess this important and current topic of burnout among all Brazilian anesthesiologists and anesthesiology residents during the COVID 19 pandemic. Would be a good statement to start the discussion. In the discussion is stated: This study provides a representative proportional sample of Brazilian anesthesiologists, according to members data previously provided by SBA However in the results it is stated that: Notably, the proportion of female members in the SBA during the study period was relatively lower, with 37.8% female and 62.2% male. Could the authors provide some clarification about this seeming discrepancy?

Response: Thank you for your detailed feedback. We have revised the introduction to better connect the examined variables to the research question and included the suggested statement at the beginning of the discussion. We have also clarified the discrepancy regarding the gender distribution by noting that while our sample is representative of the broader SBA membership, it reflects the actual gender distribution within the SBA during the study period.
