# Supplementary material for: Prevalence of burnout syndrome in Brazilian anesthesiologists during the COVID-19 pandemic: A cross-sectional survey
Source: PLoS One. 2025 Feb 18;20(2):e0313538. doi: 10.1371/journal.pone.0313538 (PMC11835280; doi:10.1371/journal.pone.0313538)
Supplement: Attachment — Submitted filename: _Response to Reviewers - 2nd Review.docx [file pone.0313538.s006.docx]

**Prevalence of Burnout Syndrome in Brazilian Anesthesiologists during the COVID-19 Pandemic: A Cross-Sectional Survey**

**Response to Reviewers – Revised Manuscript (Second Review)**

Dear Editor and Reviewer 2,

We sincerely appreciate the thoughtful feedback provided during the review process. Your valuable insights have guided us in refining and strengthening our manuscript.

In this revised version, we have carefully addressed all the points raised, including adjustments to the introduction and discussion sections, and we have further enhanced the clarity of the conclusion based on your suggestions.

We believe that these revision have significantly improved the overall quality of the work and aligned it more closely with the expectations of PLOS ONE. Thank you again for your constructive input, which has been instrumental in shaping the final version of this manuscript.

We look forward to your review of this revised submission.

Kind regards,

The authors

**Reviewer #2 (Highlighted in Yellow)**

**General Feedback:** The rebuttal and text changes are sufficient. However a few points remain which the authors should consider:

**Comment 1:** Still missing is the connection between the hypothesis in the introduction and the conclusion in the discussion; not closing the circle so to say (see 5). Added to the introduction is now: The hypothesis that burnout prevalence is higher during the pandemic. Higher compared to which numbers? The paper would gain by referencing pre-covid anaesthetist burnout prevalence, preferably from Brasil otherwise from other countries to emphasize the importance of the problem. This is done elsewhere in the paper, but a short sentence in the introduction with references would do. (eg. Something like: Burn out in anesthesiologists is an important problem, international (pre covid) anaesthetist burnout literature report prevalences of ... to ... %)

**Response 1:** Thank you for your insightful comment. We agree with your suggestion and have revised the introduction to strengthen the connection between the hypothesis and the conclusion. Specifically, we have added references to both pre-pandemic burnout prevalence among Brazilian anesthesiologists and post-pandemic international data, to better contextualize the hypothesis that burnout prevalence increased during the COVID-19 pandemic.

The revised introduction now includes pre-pandemic data from Brazil, where burnout rates ranged from 2.43% to 10.4% in small samples from specific states, alongside international post-pandemic studies reporting burnout rates among anesthesiologists as high as 30% to 60%. This contrast highlights the potential impact of the pandemic on increasing burnout prevalence and provides a stronger foundation for our hypothesis. We believe this revision aligns the introduction more closely with the discussion and conclusions.

**Comment 2:** In your answer to comment 2 you state: “The prevalence of burnout found in our study did not show a significant increase”. Related to above, the question here is: increase compared to which numbers/ what/ when? See 3.

**Response 2:**  Thank you for this important question. Initially, we based our comparison on a previous study from our research group, "Impact of the COVID-19 Pandemic on the Prevalence of Burnout among Residents in Orthopedics," which was conducted in a small sample of orthopedic residents. This study found no significant increase in burnout prevalence or severity before and during the pandemic. However, after reflecting on your comment and reviewing additional literature, we realize that it is crucial to focus on the specific context of anesthesiology during the pandemic, where burnout has been shown to increase significantly.

In light of this, we revised our approach, incorporating relevant studies on anesthesiologists that highlight the heightened risk and prevalence of burnout during the COVID-19 pandemic. The national and international data, along with our findings, suggest a clear increase in burnout among anesthesiologists, which may not be generalizable from other specialties like orthopedics. We have updated the manuscript to reflect this change in perspective and clarify the increase in burnout specifically within anesthesiology during the pandemic, supported by additional studies.

**Comment 3:** In your answer to comment 4 you state: Therefore, a direct comparison with figures from before the pandemic for Brazilian anesthesiologists is not feasible due to the lack of specific pre-pandemic studies within this population. Are you sure there have been no earlier studies into Brazilian anesthetist burnout rates, as you state in your answer to comment 4? How about: Magalhães, Edno, et al. "Prevalence of burnout syndrome among anesthesiologists in the Federal District." Revista brasileira de anestesiologia 65 (2015): 104-110. Coincidentally, the first sentence of this paper closely resembles the first sentence of your paper. Also: Govêia, Catia Sousa, et al. "Association between burnout syndrome and anxiety in residents and anesthesiologists of the Federal District." Revista brasileira de anestesiologia 68 (2018): 442-446.)

**Response 3:** Thank you for bringing these studies to our attention. Initially, we did not include these studies in our work because they focus on specific, localized samples (residents and anesthesiologists in the Federal District), and our study aims to provide a more comprehensive analysis of the situation across Brazil. However, we fully agree that these are the only published pre-pandemic sources on the prevalence of burnout among anesthesiologists in Brazil, and it is essential to include and compare them with the broader national context during the COVID-19 pandemic.

We have now incorporated the studies by Magalhães et al. (2015) and Govêia et al. (2018) into both the introduction and discussion sections of the manuscript. These studies, though based on small and localized samples, provide valuable pre-pandemic data on Brazilian anesthesiologists, with burnout prevalence ranging from 2.43% to 10.4%. These findings allow us to make more meaningful comparisons with our post-pandemic data, which shows a marked increase in burnout prevalence, and thus strengthens the conclusions of our study.

**Comment 4:** Concerning reference 20 consider also referencing this survey from Brasil, in residents anaesthesiology. Pietroski dos Santos, Natanael, et al. "Burnout risk among anesthesiology residents in Brazil during the second wave of COVID-19: a cross-sectional survey." Brazilian Journal of Anesthesiology 73.1 (2022): 120-122.

**Response 4:** Thank you for recommending this relevant reference. We have added the study by Pietroski dos Santos et al. (2022) to our manuscript. This study provides critical insight into the burnout risk among anesthesiology residents during the second wave of COVID-19, with findings showing that 73.2% of residents were at risk of burnout and 57.1% were at high risk.

Although this study used a different instrument (the Oldenburg Burnout Inventory, rather than the Maslach Burnout Inventory), it still offers valuable data on the increasing burnout risk during the pandemic. We have noted the methodological differences between our study and this reference in the discussion section, emphasizing that despite the different measurement tools, the consistent findings of increased burnout risk in both studies underline the severity of the issue among anesthesiology professionals during the pandemic.

**Comment 5:** In your conclusion: This study represents a comprehensive assessment of burnout among all Brazilian anesthesiologists during the COVID-19 pandemic, highlighting a high prevalence of burnout and its associate risk factors. This conclusion is in line with your findings. But: In order to relate to your hypothesis and in line with your findings and discussion, perhaps I would recommend to add: However, burnout rates (in Brazilian anaesthetists) seem not to be higher during the pandemic compared to burnout rates pre-pandemic. (internationally or in Brasil.

**Response 5:** Thank you for your insightful suggestion. We agree that incorporating a comparison between pre-pandemic and pandemic burnout rates will provide a more nuanced understanding of the findings. While we acknowledge the elevated burnout rates observed during the pandemic, the available pre-pandemic studies in Brazil show similarly high levels of burnout, suggesting that the pandemic may not have led to a significant increase in burnout rates among anesthesiologists. We have revised the conclusion accordingly to reflect this point and align it with our discussion and findings.
